# Effects of High-Intensity Interval Training on Executive Functions in College Students: Evidence from Different Doses

**DOI:** 10.3390/brainsci13040571

**Published:** 2023-03-28

**Authors:** Xiaomei Wang, Zhigang Liu, Huanyu Zhang, Chaoxin Ji

**Affiliations:** 1 Physical Education Department, Northeastern University, Shenyang 110819, China; wangxiaomei@pe.neu.edu.cn; 2 Aviation Physical Education Department, Aviation University of Air Force, Changchun 130022, China

**Keywords:** high-intensity interval training, college students, executive functions, different doses

## Abstract

Background: Different doses of high-intensity interval training (HIIT) may affect individuals’ executive functions (EF). In this study, low-dose HIIT and moderate-dose HIIT were used to explore different doses of HIIT in terms of the impact on the EF of college students. Methods: All the participants were randomly divided into three groups. One group was not assigned any HIIT, which was called the control group. One group was assigned one session of HIIT/week, which was called the low-dose group. The last group was assigned wo sessions of HIIT/week, which was called the moderate-dose group. All groups were subjected to EF measurements. The first measurement comprised an EF a baseline measurement (Time 1) before the experiment began; the second measurement was taken (Time 2) after 6 weeks; the third measurement was taken (Time 3) after 12 weeks. Results: Time 1: We found that there was no significant difference in EF among the groups (*p* > 0.05). Time 2: The moderate-dose group and low-dose group improved in terms of EF. However, the improvement effect was different, and the improvement effect of the moderate-dose group EF was better than that of the low-dose group. The second measurement, EF was better for all exercise groups than for the control group. Inhibition test (reaction time: 3.97–8.24%, *p* < 0.05, effect size: 0.413); cognitive flexibility (accuracy: 6.66–7.32%, *p* < 0.05, effect size: 0.203; reaction time: 5.55–7.49%, *p* < 0.05, effect size: 0.521); working memory (accuracy: 4.05–4.69%, *p* < 0.05, effect size: 0.515; reaction time: 2.73–5.42%, *p* < 0.05, effect size: 0.430). Time 3: the moderate-dose group and low-dose group showed a downward trend in terms of EF. Conclusion: Low-dose HIIT and moderate-dose HIIT improved the EF in college students, but moderate-dose HIIT was better. This study suggests that moderate-dose HIIT should be adopted to improve the EF in college students.

## 1. Introduction

Among many cognitive functions, executive functions (EF) are the most crucial expression of cognitive function. EF comprise the process of helping individuals to control their behaviors [1]. EF comprise a psychological process of problem-solving, which enables individuals to coordinate various cognitive resources and manage cognitive systems to complete various processing tasks, while completing complex cognitive tasks [2]. EF mainly consist of inhibition, cognitive flexibility, and working memory [3]. Inhibition refers to the ability to control attention, ideas, and emotions; cognitive flexibility is the ability to change one’s view of something and adapt to new circumstances; working memory means that external data can be retained briefly in an individual [4,5,6]. The sub-functions of the EF are interrelated. The research indicated that EF have an essential influence on the mental health, academic achievement, and behavior of individuals [7,8]. Because EF impact every aspect of individuals’ lives, people have done more in-depth research on EF. How to improve EF has been a topical issue for many years.

There is a beneficial role of physical exercise in the general health of adults according to the latest guidelines by the World Health Organization [9] and the American College of Sports Medicine [10]. The plasticity of EF is one of its characteristics. Some studies have determined that exercise can effectively improve the EF of individuals. For example, Chou pointed out that when the Stroop task was conducted immediately after the acute resistance training and 40 min later, there was not significant difference in the test scores between the immediate test and the 40 min later test, but both were better than the Stroop test scores before the acute resistance training [11]. It has shown that acute exercise can effectively improve individuals’ inhibition. There is also a significant relationship between EF and exercise types. Park’s study determined that moderate-intensity aerobic exercise can significantly improve the EF of the elderly in the community [12]. Many studies have pointed out the influence of exercise’s intensity and frequency on EF. Generally speaking, the greater the intensity of exercise and the higher the exercise frequency, the more improvements are observed in EF [13,14,15]. Moeller’s study has shown that exercise intensity can effectively affect the EF of individuals. In Moeller’s study, moderate-intensity physical exercise had a positive effect on inhibition control, while high-intensity physical exercise had no effect on inhibition control [16].

In recent years, high-intensity interval training (HIIT) has gradually stepped into people’s daily fitness activities. HIIT is highly respected by fitness people because of its high exercise efficiency and short time consumption. HΙΙΤ is popular in the health and fitness industry at a global level, according to the latest report published by the American College of Sports Medicine [17]. According to the current research viewpoint, HIIT can effectively improve individual EF. Hsieh’s study has shown that HIIT has beneficial effects on EF in children, adults, and the elderly [18]. It was proven that HIIT can improve the EF of individuals of different ages. It also shows that the EF of individuals of all ages was plastic. Zhu compared the effects of HIIT and moderate-intensity continuous exercise on the EF of healthy young men. The study found that both types of exercise can improve individual EF, but the extent of improvement was slightly different. In Zhu’s study, HIIT improved individual EF better than moderate-intensity continuous exercise [19]. However, some studies have shown that HIIT cannot effectively improve the EF of individuals, and that exercise even has harmful effects on EF. Anders’s research points out that after HIIT, the accuracy of mathematical processing tasks and reaction time of subjects was significantly reduced [20]. It has been shown that HIIT has a negative impact on individual’s EF. The possible cause of HIIT leading to the decline of individual EF is fatigue. After HIIT, the individual is in a state of fatigue. If EF is measured during this period, the test results may be influenced due to fatigue. It is also possible that the results are different in these studies because of the measurement methods. Srinivas’s research has shown that different exercise types have different improvement effects on EF, and other measurement methods will also affect the test results [21]. Moreover, some studies have pointed out that HIIT will reduce EF. For example, Costello’s research pointed out that after several days of HIIT, there was a reduction in the EF of rugby players, and they suggested this decrease of EF might lead to an increase in the probability of injuries in sports [22]. 

Therefore, to resolve the above disputes, this study asked college students to practice HIIT, and then tested their EF, in order to observe the impact of HIIT on the EF of college students. In this study, we divided college students into three groups. The first group did not practice any form of HIIT (control group), the second group practiced HIIT once a week (low dose group), and the third group practiced HIIT twice a week (moderate dose group). We discuss the influence of different doses of HIIT on college students’ EF. We hypothesized that HIIT would have a beneficial effect on the EF of college students, but that impact would be different. When compared with low-dose HIIT, moderate-dose HIIT can improve the EF of college students to a greater extent.

## 2. Materials and Methods

### 2.1. Participants

When recruiting subjects, we first used G*Power (3.1.9.7) to conduct a power analysis. We selected the average effect size of 0.25; the alpha of 0.05; the power of 0.85; the number of measures of 3; the number of groups of 3. After calculation, at least 63 subjects were needed to meet the needs of the analysis. Considering the dropout rate of about 20%, at least 76 subjects needed to be recruited.

**Figure 1:**
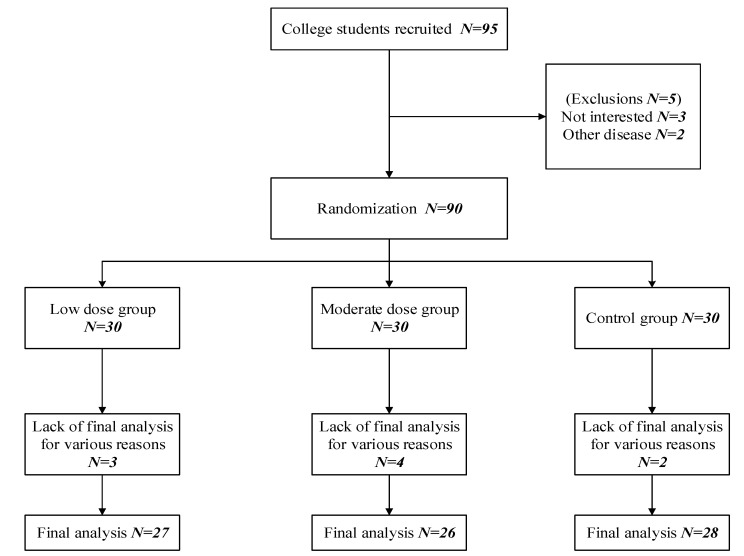
Participant flowchart across the study.

In this study, the subjects were recruited from a university (the recruitment college students were undergraduates). The inclusion criteria of the study were as follows: (1) Subjects who were physically and psychologically healthy; (2) no major traumatic events have been experienced in the last year; (3) subjects who were right-handed; (4) all subjects had to sign a statement of informed consent; (5) no other diseases were found in physical examination. This study was approved by the Ethics Committee of Northeastern University. A total of 95 college students were recruited in this study. We excluded college students who did not meet the research standards and those who did not participate in the test for various reasons. Finally, a total of 81 college students participated in the study, including 51 males and 30 females. The related screening flow chart is shown in Figure 1.

### 2.2. Study Methodology

**Figure 2:**
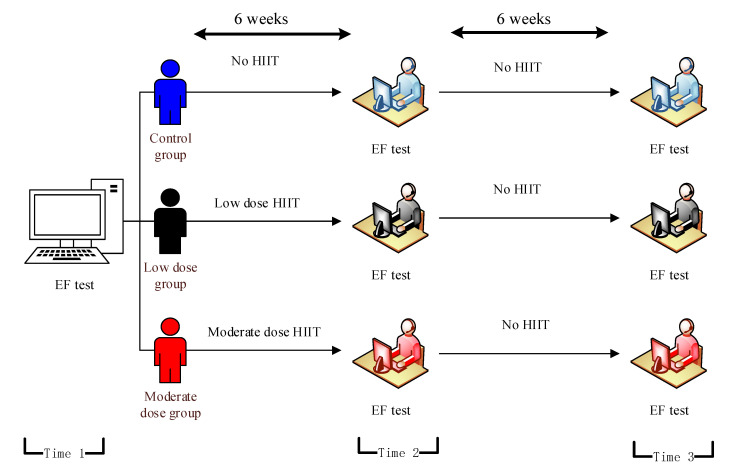
Experimental flow chart.

The study was randomized, crossover-designed, and included four laboratory visits. The first laboratory visit was implemented in order to screen the subjects, mainly including the collection of basic information about the subjects, so that the subjects were familiar with the test procedures of EF. In this process, we let each subject perform the EF test separately until the subjects were familiar with the EF test process, in order to minimize the learning effect of the subjects in future tests. After the formal experiment began, the subjects measured their EF three times. The first measurement was made one day before the formal experiment, the second after the experiment, and the third after the second measurement without any intervention, and the measurement was made again six weeks later. A total of 90 subjects were randomly divided into three groups using an online resource (http://www.randomization.com, accessed on 13 February 2022). The first group was called the low-dose group, and the number of subjects in the low-dose group was 30, including 19 males and 11 females; the second group was called the moderate-dose group, and the number of subjects in the moderate dose group was 30, including 18 males and 12 females; the third group was called the control group, and the number of subjects in the control group was 30, including 16 males and 14 females. One session of HIIT/week was used for the low-dose group; 2 sessions of HIIT/week were used for the moderate dose group; the control group did not use for any form of intervention, and kept their normal living conditions. The Borg RPE scale was used to monitor the exercise intensity of subjects. Recumbent bikes (STAR TRAC, Irvine, CA, USA) were used for HIIT, and the real-time heart rate of the subjects was displayed on the recumbent bike. The HIIT program was as follows: first, the subjects warmed up for 5 min, and after finishing the warm-up, they entered the experiment formally. Subjects were asked to ride as quickly as they could on the recumbent bike for 60 s, then slow down and ride slowly on the recumbent bike for 60 s. Riding rapidly for 60 s and riding slowly for 60 s were movement processes, and the whole process was repeated 5 times. Each experiment lasted for 10 min. After the experiment, 5 min was dedicated to relaxation activities for the subjects. The experiment lasted for 6 weeks, during which the low-dose group trained 6 times and the moderate-dose group trained 12 times in total. During the experiment, the unqualified subjects were eliminated over time. The experimental flow is shown in Figure 2.

### 2.3. EF Test

Inhibition test. Stroop task: the Stroop task was used to measure response inhibition [23]. The Stroop task was divided into congruent and incongruent tasks. Before each trial, a white “+” fixation point was presented in the middle of the computer screen for 500 ms, and then for 1500 ms, colored words (red, yellow, green and blue) were randomly presented in the center of the screen. In the congruent test, the subjects were presented with one of four stimuli, red, yellow, green and blue, and the colors presented each time were consistent with the meaning of the words, so the subjects were required to press a key corresponding to the colors of the word. In the incongruent test, the same stimulus was presented, but the color presented each time was inconsistent with the meaning of the word, so the subjects were required to press a key corresponding to the the color of the word. The congruent and incongruent tests accounted for 25% and 75% of the total number of trials. This study used the reaction time and accuracy of the incongruent trials as the main dependent variables for this task.

Cognitive Flexibility. More_odd shifting task: The More_odd shifting task can be applied to measure cognitive flexibility [24]. More_odd shifting mainly involves digital conversion tasks to evaluate individual cognitive flexibility. The More_odd shifting task consisted of a series of numbers that were gradually presented in the center of the computer screen. The presentation time of the numbers was 2000 ms, and the stimulation interval was 1000 ms. The subjects were required to judge the numbers from 1 to 9 (excluding 5). There were three kinds of number judgment tasks. A: odd and even number judgment. When the number is red, judge whether it is an odd or even number. Press A for an odd number and L for even numbers. B: Number size judgment. When the number appears green, judge whether the number is greater than 5 or less than 5; press A for less than 5, and press L for greater than 5. C: Mixed digital judgment. When the numbers ae red, odd and even numbers are judged; when the number appears green, the number’s size is judged. The formal test was divided into 6 segments, in which each form of digital judgment task was 2 segments, each segment was 30 times, and the interval between each segment was 20 s. This study used the reaction time and accuracy of the mixed digital judgment as the main dependent variables for this task. 

Working memory. The 2-back task was used to measure working memory [25]. Nine discontinuous sequences of 9 digits (1–9) were used as experimental stimuli. Each sequence of digits had 20 trials, and each stimulus was presented for 500 ms. After the task starts, a digit was randomly presented in the middle of the screen, and the subjects were required to compare the current digit with the second digit in front. If it was the same, they pressed A, and if it was different, they pressed L. There were 12 trials at the beginning of the experiment. After the experiment officially started, there were 120 trials in total. The measurement indexes were accuracy and the reaction time.

### 2.4. Statistical Analysis

SPSS 26.0 was used for statistical analysis. Per protocol analysis was used. All means and standard deviations (SD) were statistically analyzed using standardized statistical methods. The Shapiro–Wilk test and visual inspection of residuals were used to verify the assumption of normality. Mauchly was used for sphericity test, but if the results of sphericity test were not consistent, the Greenhouse–Geisser test was used for analysis. The dependent variables of measurement were independent, and included inhibition, cognitive flexibility and working memory. Estimates are expressed as 95% confidence intervals (CI). Partial Eta squared ($\eta_{p}^{2}$) was used to calculate effect sizes for significant main effects and their interactions. A mixed-effect analysis of the variance 3 (type: low-dose HIIT, moderate-dose HIIT, no HIIT) × 3 (measurement: time 1, time 2, time 3) model was used to count the subjects’ inhibition, cognitive flexibility and working memory.

## 3. Results

### 3.1. Subjects Characteristics

A total of 90 subjects participated in the experimental test, and 81 subjects were eventually included in the statistical results, with a dropout rate of 10%. In the final statistics of the subjects, we calculated the average attendance rate of each group; the average attendance rate of the low-dose group was 94.50%, and the average attendance rate of modern dose group was 94.33%. The attendance rate meets the needs of this study. We used an exercise cardiorespiratory fitness testing system (Smax58ce, Highermed, Nanjing, China) to measure VO_2MAX_, and used the International Physical Activity Questionnaire (IPAQ) to record the subject’s weekly physical activity. We analyzed the subjects characteristics, mainly in terms of their age (F (2, 78) = 0.703, *p* > 0.05), gender (χ^2^ (2) = 0.891, *p* = 0.641), body mass index (F (2, 78) = 0.410, *p* > 0.05), VO_2MAX_ (F (2, 78) = 0.501, *p* > 0.05), physical activity (F (2, 78) = 0.405, *p* > 0.05), and resting heart rate (F (2, 78) = 0.312, *p* > 0.05). It was found that there were no significant differences in the essential characteristics of the three groups, which shows that the essential characteristics of the three groups had good consistency (Table 1).

### 3.2. Effects of Low vs. Moderate Dose HIIT on Inhibition 

**Figure 3:**
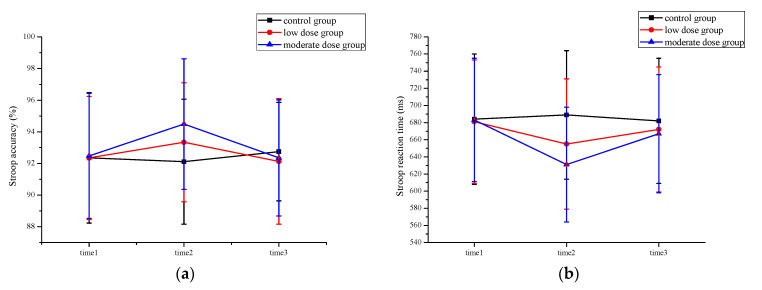
The trend of inhibition of the three groups of college students ((**a**) inhibition accuracy; (**b**) inhibition reaction time).

In order to investigate the effect of different doses of HIIT on inhibition, through a mixed-effect analysis of variance, it was found that the main effect of measurement time on the accuracy of inhibition was not significant, F (2, 77) = 22.30, *p* = 0.221, $\;\eta_{p}^{2}=0.021$. There was no significant difference in the accuracy of the three measurements (Figure 3a and Table S1). The interaction effect between accuracy and different doses of HIIT was not significant, F (4, 156) = 1.04, *p* = 0.113, $\;\eta_{p}^{2}=0.015$. It was found that the main effect of measurement time on the reaction time of inhibition was significant, F (2, 77) = 137.90, *p* < 0.001, $\;\eta_{p}^{2}=0.498$. The reaction time of the second measurement was obviously shorter than that of the third measurement and the first measurement. The interaction effect between reaction time and different doses of HIIT was significant, F (4, 156) = 75.49, *p* < 0.001, $\;\eta_{p}^{2}=0.252$. The second measurement, EF, was showed better values in all exercise groups when compared to the change seen in the control group. The reaction time: 3.97–8.24%, *p* < 0.05, 95% CI: [602.12–732.28]–[598.19–709.56], effect size: 0.413 (Tables S1 and S2). Between-groups comparison: There were no significant differences between the three groups for the first measurement, but there was a significant difference between the three groups for the second measurement and the third measurement. Intra-group comparison: The second measurement of reaction time in the low-dose group and moderate-dose group was shorter than the third measurement, and both were shorter than the first measurement (Figure 3b and Table S1).

### 3.3. Effects of Low vs. Moderate Dose HIIT on Cognitive Flexibility 

**Figure 4:**
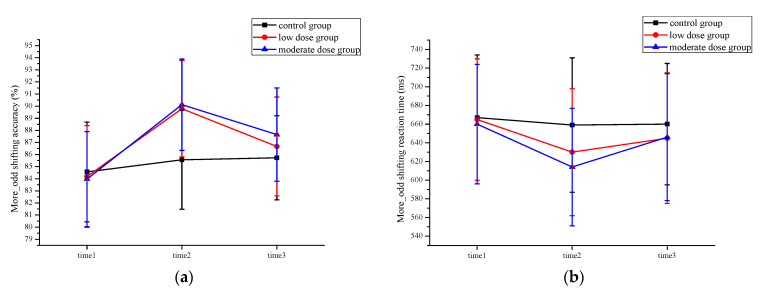
The cognitive flexibility of the three groups of college students ((**a**) More_odd shifting accuracy; (**b**) More_odd shifting reaction time).

In order to investigate the effect of different doses of HIIT on cognitive flexibility, through mixed-effect analysis of variance, it was found that the main effect of measurement time on the accuracy of cognitive flexibility was significant, F (2, 77) = 81.24, *p* < 0.001, $\;\eta_{p}^{2}=0.312$. There were no significant differences in cognitive flexibility accuracy between the second and third measurements of the low-dose group and moderate-dose group, but both were significantly higher than the first measurements. The interaction effect between accuracy and different doses of HIIT was significant, F (4, 156) = 32.78, *p* < 0.001, $\;\eta_{p}^{2}=0.115$. The second measurement of EF was better in all exercise groups than that of the control group. The accuracy was 6.66–7.32%, *p* < 0.05, 95% CI: [80.12–88.68]–[82.13–87.49], and the effect size was 0.203 (Tables S1 and S2). Between-groups comparison: The accuracy of the second and third measurements of the moderate-dose group was higher than that of the low-dose group. Intra-group comparison: The accuracies of the second measurement and the third measurement of the moderate-dose group and low-dose group were higher than the accuracy of the first measurement (Figure 4a and Table S1). It was found that the main effect of measurement time on the reaction time of cognitive flexibility was significant, F (2, 77) = 79.31, *p* < 0.001, $\;\eta_{p}^{2}=0.253$. The reaction time of the second measurement was obviously shorter than that of the third measurement and the first measurement. The interaction effect between reaction time and different doses of HIIT was significant, F (4, 156) = 11.31, *p* < 0.001, $\;\eta_{p}^{2}=0.104$. The second measurement of EF was better in all exercise groups than that of the control group. The reaction time was 5.55–7.49%, *p* < 0.05, 95% CI: [600.73–722.56]–[587.12–713.46], and the effect size was 0.521 (Tables S1 and S2). Between-groups comparison: There were no significant differences between the three groups regarding the first measurement, but there were significant differences between the three groups regarding the second measurement and the third measurement. Intra-group comparison: The second measurements of the reaction time in the low-dose group and moderate-dose group were shorter than the third measurement of reaction time, and both were shorter than the first measurement (Figure 4b and Table S1).

### 3.4. Effects of Low vs. Moderate Dose HIIT on Working Memory 

**Figure 5:**
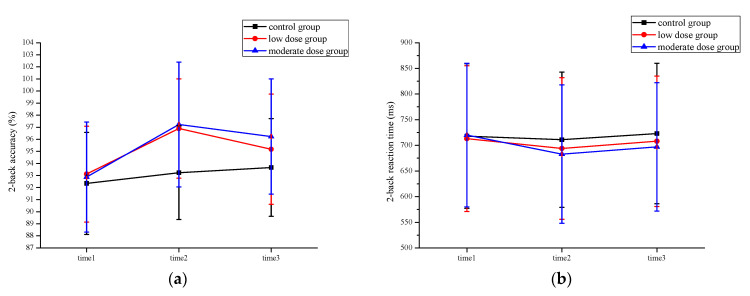
The trend of working memory of the three groups of college students ((**a**) 2-back accuracy; (**b**) 2-back reaction time).

In order to investigate the effect of different doses of HIIT on working memory, through mixed-effect analysis of variance, it was found that the main effect of measurement time on the accuracy of working memory was significant, F (2, 77) = 148.90, *p* < 0.001, $\;\eta_{p}^{2}=0.313$. There was no significant difference in working memory accuracy between the second and third measurements of the low-dose group and moderate-dose group, but both were significantly higher than the first measurements. The interaction effect between accuracy and different doses of HIIT was significant, F (4, 156) = 81.23, *p* < 0.001, $\;\eta_{p}^{2}=0.253$. The second measurement of EF was better for all exercise groups than for the control group. The accuracy was 4.05–4.69%, *p* < 0.05, 95% CI: [88.05–96.54]–[89.46–95.43], and the effect size was 0.515 (Tables S1 and S2). Between-groups comparison: The accuracies of the second and third measurements of the moderate-dose group were higher than those of the low-dose group. Intra-group comparison: The accuracies of the second measurement and the third measurement of the moderate-dose group and low-dose group were higher than the accuracy of the first measurement (Figure 5a and Table S1). It was found that the effect of measurement time on the reaction time of working memory was significant, F (2, 77) = 131.23, *p* < 0.001, $\;\eta_{p}^{2}=0.335$. The second measurement of reaction time was obviously shorter than that of the third measurement and the first measurement. The interaction effect between reaction time and different doses of HIIT was significant, F (4, 156) = 73.46, *p* < 0.001, $\;\eta_{p}^{2}=0.211$. The second measurement of EF was better for all exercise groups for the change in the control group. The reaction time was 2.73–5.42%, *p* < 0.05, 95% CI: [579.59–830.23]–[564.12–820.32], and the effect size was 0.430 (Tables S1 and S2). Between-groups comparison: There were no significant differences between the three groups in the first measurement, but there was a significant difference between the three groups in terms of the second measurement and the third measurement. Intra-group comparison: The second measurements of the reaction time of the low-dose group and moderate-dose group were shorter than the third measurement, and both were shorter than the first measurement (Figure 5b and Table S1). 

## 4. Discussion

This study explored the effect of different doses of HIIT on the EF of college students. Studies have shown that both low- and moderate-dose HIIT can improve the EF of college students. After measuring the EF of college students, it was found that different doses of HIIT had different effects on the improvement of EF. Generally speaking, the impact of moderate-dose HIIT on the EF of college students was better than that of low-dose HIIT.

Many studies have shown that HIIT had a beneficial impact on EF. Different doses of HIIT had different effects on each sub-function of the EF. This study has shown that the improvement of the EF of subjects subjected to HIIT twice a week was better than for those subjected to HIIT once a week. In this study, we can see that-low and moderate-dose HIIT effects the inhibition accuracy, but this was not significant. In terms of the reaction time to the inhibition, it was found that the reaction time of the moderate-dose group was shorter than that of the low-dose group. This has shown that the effect of moderate-dose HIIT on college students’ inhibition was better. As for the influence of physical exercise on inhibition, many studies believe that physical exercise can improve the inhibition of individuals. Kao’s research has shown that just 20 min of aerobic exercise can improve the inhibition control of individuals [26]. Nouchi used the Stroop task to measure inhibition in middle-aged and older women. The study found that 30 min of aerobic training significantly improved inhibition in middle-aged and older women [27]. Kao and Nouchi’s research has shown that a single exercise session can effectively improve an individual’s inhibition control. One study showed that a single exercise session/week improved musculoskeletal fitness, and demonstrated a step-wise improvement with two and three sessions/week, suggesting a dose-dependent response [28]. Chang used the Stroop task to investigate the influence of different training times on inhibition. It was found that in those training three times across 4 days, moderate- and high-intensity acute exercise lasting 20 min had the most significant influence on the inhibition of middle-aged and older adults, while moderate- and high-intensity acute exercise lasting 10 min and 45 min had little impact on their inhibition [29]. The above studies have shown that exercise time has a particular impact on individual’s inhibition. In this study, different doses of HIIT were used for college students. Our research has shown that the effect of moderate-dose HIIT was better than that of low-dose HIIT.

For HIIT to improve the cognitive flexibility of college students, this study has shown that the effect of moderate-dose HIIT on cognitive flexibility was better than low-dose HIIT. Shukla’s research has shown that a single, 20 min aerobic exercise can effectively improve individual cognitive flexibility [30]. This study has demonstrated that different doses of HIIT can effectively improve the cognitive flexibility of college students. Mekari’s study has shown that cognitive flexibility improved in older adults after 6 weeks of HIIT to a greater extent than those participating in moderate-intensity continuous training and resistance training [31]. Our research was consistent with Mekari’s research. After 6 weeks of HIIT, college students’ cognitive flexibility had improved. Netz’s research has shown that a single aerobic exercise session can improve cognitive flexibility. However, after 1 h, cognitive flexibility decreased, indicating that a single aerobic exercise cannot promote this improved cognitive flexibility for a long time [32]. We found that after 6 weeks of HIIT, cognitive flexibility was improved, but after 6 weeks of stopping HIIT, we found that cognitive flexibility remained at a high level. Our research has shown that HIIT has a good effect on improving college students’ cognitive flexibility. The mechanism of exercise improving cognitive flexibility can be explained as follows. Event-related potential (ERP) was used to measurement cognitive flexibility, which demonstrated that acute aerobic exercise can effectively improve cognitive flexibility [33]. It has shown that physical exercise could promote the change of a particular type of neuron in the brain, which then could lead to the improvement of cognitive flexibility. Brockett’s research found that running can improve cognitive flexibility. Through further analysis, it was found that running mainly enhances synapses and dendrites in several brain regions involved in cognition, which proves that physical exercise induces plasticity in neurons [34]. Therefore, physical exercise can improve cognitive flexibility, mainly because neurons have plasticity, and physical exercise can effectively improve the plasticity of neurons, so that cognitive flexibility can be effectively improved.

For HIIT to improve college students’ working memory, this study found that different doses of HIIT have different improvement effects on working memory. HIIT could significantly improve the accuracy and reaction time of working memory. Moreover, moderate-dose HIIT improves working memory better than low-dose HIIT. Wheeler’s study has shown that moderate-intensity exercise can effectively improve the working memory of the elderly [35]. It has shown that exercise can effectively improve individual working memory. As for the types of exercise, Wen’s study has shown that resistance training, coordination training, and football training can effectively enhance children’s working memory, and there is not significant difference among the three kinds of exercise [36]. Wen’s research has shown that exercise types have the same improvement effect on working memory. However, for different exercise intensities, there are varied results. Wilke’s research has shown that high-intensity training improves working memory better than walking [37]. Mou’s research has shown that both HIIT and moderate-intensity continuous training can improve college students’ working memory, but there were differences in terms of these effects. If the college students who exercise more at ordinary times were more suitable for HIIT, the college students who exercise less at ordinary times were more suitable for moderate-intensity continuous training [38]. Mou’s research has shown that the effect of exercise intensity on the improvement of individual working memory was different. As to why HIIT can significantly improve working memory, Kao compared HIIT with moderate-intensity continuous exercise and found that both HIIT and moderate-intensity exercise can improve working memory, but only HIIT can improve the processing speed in terms of memory retrieval [39]. The mechanism of improving working memory through physical exercise has been explained in other works. ERP was used to prove that spatial working memory is improved during exercise [40]. Drollette determined that HIIT may facilitate improvements in underlying mental operations that are responsible for temporal stability in cognitive and neurocognitive function [41]. Therefore, the mechanism of improving working memory by physical exercise may be via the reconstruction of neural units related to working memory, which leads to an improvement in working memory.

This study has shown that different doses of HIIT have different effects on each sub-function of EF. Generally speaking, moderate-dose HIIT has a better effect on improving the EF of college students. We applied HIIT for the intervention and the study was actionable. Therefore, we suggest that college students should be allowed to perform HIIT twice a week, so that their improved EF can continue. However, this study also has limitations. First, this study was aimed at a single population, only college students, and there was no comparative study of different age groups. Secondly, because the subjects were college students, there may be some other exercise behaviors during the experiment. Although we did not carry out activities outside the experiment, it was difficult to completely track the exercise situation of college students, so this aspect also has certain limitations. In future research, it was necessary to conduct in-depth research on these existing limitations.

## 5. Conclusions

This study demonstrated the effectiveness of HIIT in improving the EF of college students. However, different doses of HIIT had different effects on college students’ EF. Compared with low dose HIIT, moderate-dose HIIT had a better effect on improving the EF of college students. However, once HIIT was stopped, although the EF of college students was higher than that at the first measurement, the EF of college students still showed a downward trend. It has been shown that long-term exercise may be an essential factor in maintaining EF. In future studies, the factors influencing the improvement of EF could be further analyzed to distinguish the role of covariates.

## Figures and Tables

**Table 1:** Subjects’ characteristics (M ± SD).

Characteristics	Virtual Training Group	Physical Exercise Group	Control Group	*p*-Value
Number	27	26	28	-
Age (years)	22.55 ± 2.76	21.98 ± 2.25	22.79 ± 2.36	0.647
Gender (male/female)	17/10	17/9	15/13	0.641
BMI (kg/m^2^)	20.39 ± 2.77	21.32 ± 4.82	20.94 ± 2.01	0.301
VO_2MAX_ (mL/kg/min)	39.46 ± 4.59	37.45 ± 5.67	36.95 ± 6.08	0.103
Physical activity (min/week)	58.49 ± 18.36	52.49 ± 18.35	55.38 ± 17.64	0.115
Resting HR (bpm)	77.32 ± 9.35	77.8 ± 10.34	77.8 ± 10.34	0.201

Abbreviations: M, mean; SD, standard deviation; BMI, body mass index; HR, heart rate; VO_2MAX_, maximal oxygen consumption.

## Data Availability

The data presented in this study are available on request from the corresponding author. The data are not publicly available due to privacy reasons.

## Supplementary Materials

The following supporting information can be downloaded at: https://www.mdpi.com/article/10.3390/brainsci13040571/s1, Table S1: Results of measured variables; Table S2: Statistical information for all comparisons.
