# Supplementary material for: Effects of High-Intensity Interval Training on Executive Functions in College Students: Evidence from Different Doses
Source: Brain Sci. 2023 Mar 28;13(4):571. doi: 10.3390/brainsci13040571 (PMC10136687; doi:10.3390/brainsci13040571)
Supplement: Supplementary file 1 [file brainsci-13-00571-s001.zip › Table S1. Results of measured variables.pdf]

Table S1 Results of measured variables

**Variables**	**time**	**Control group**		**Low dose group**		**Moderate dose group**	
		**M**	**SD**	**M**	**SD**	**M**	**SD**
Inhibition accuracy (%)	Time 1	92.35	4.12	92.35	3.89	92.48	3.95
	Time 2	92.11	3.95	93.34	3.76	94.49	4.13
	Time 3	92.75	3.11	92.13	3.97	92.35	3.67
Inhibition reaction time (ms)	Time 1	684.36	76.68	681.45	72.32	683.67	72.32
	Time 2	689.54	75.92	655.43	76.87	631.89	67.79
	Time 3	682.67	73.11	672.49	73.89	667.93	69.72
Cognitive flexibility accuracy (%)	Time 1	84.56	4.12	84.18	4.21	83.97	3.93
	Time 2	85.57	4.09	89.79	3.99	90.12	3.78
	Time 3	85.73	3.48	86.67	4.08	87.65	3.86
Cognitive flexibility accuracy (ms)	Time 1	667.47	67.83	665.65	65.43	660.78	64.58
	Time 2	659.54	72.56	630.12	68.16	614.65	63.43
	Time 3	660.43	65.13	645.35	70.12	646.57	68.57
Working memory accuracy (%)	Time 1	92.35	4.23	93.12	3.97	92.87	4.56
	Time 2	93.24	3.89	96.89	4.11	97.23	5.17
	Time 3	93.67	4.05	95.18	4.56	96.23	4.77
Working memory accuracy (ms)	Time 1	718.43	141.56	713.78	142.68	720.54	140.73
	Time 2	711.58	132.58	694.57	138.42	683.76	135.21
	Time 3	723.23	137.32	708.78	127.63	697.92	125.31

Abbreviations: M, mean; SD, standard deviation
