# Supplementary material for: Effects of High-Intensity Interval Training on Executive Functions in College Students: Evidence from Different Doses
Source: Brain Sci. 2023 Mar 28;13(4):571. doi: 10.3390/brainsci13040571 (PMC10136687; doi:10.3390/brainsci13040571)
Supplement: Supplementary file 1 [file brainsci-13-00571-s001.zip › Table S2. Statistical information for all comparisons..pdf]

Table S2. Statistical information for all comparisons.

Time Point	Group		95% Confidence Interval		Effect Size
Inhibition (accuracy)					
Multiple Comparisons					
Time 2	low dose group	Control group	88.49	95.73	0.012
	moderate dose group	Control group	88.34	96.00	
Time 3	low dose group	Control group	87.50	95.74	0.011
	moderate dose group	Control group	88.49	95.77	
Pairwise Comparisons					
Time 2 vs Time 1			87.02	96.88	
Time 3 vs Time 1	low dose group		88.41	96.13	0.013
Time 3 vs Time 2			89.07	96.61	
Time 2 vs Time 1			88.68	97.50	
Time 3 vs Time 1	moderate dose group		87.38	96.24	0.015
Time 3 vs Time 2			88.58	97.46	
Inhibition (reaction time)					
Multiple Comparisons					
Time 2	low dose group	Control group	602.12	732.28	0.413
	moderate dose group	Control group	598.19	709.56	
Time 3	low dose group	Control group	617.48	712.83	0.211
	moderate dose group	Control group	619.76	718.56	
Pairwise Comparisons					
Time 2 vs Time 1			613.46	723.59	
Time 3 vs Time 1	low dose group		621.38	714.32	0.311
Time 3 vs Time 2			615.73	721.67	
Time 2 vs Time 1			589.56	719.38	
Time 3 vs Time 1	moderate dose group		605.49	720.43	0.513
Time 3 vs Time 2			598.90	723.45	
Cognitive Flexibility (accuracy)					
Multiple Comparisons					
Time 2	low dose group	Control group	80.12	88.68	0.203

	moderate dose group	Control group	82.13	87.49	
Time 3	low dose group	Control group	80.43	86.54	0.102
	moderate dose group	Control group	81.56	87.32	
Pairwise Comparisons					
Time 2 vs Time 1	low dose group		82.15	87.36	0.316
Time 3 vs Time 1			80.56	86.54	
Time 3 vs Time 2			81.15	85.13	
Time 2 vs Time 1	moderate dose group		83.01	87.60	0.472
Time 3 vs Time 1			81.11	87.67	
Time 3 vs Time 2			82.95	87.32	
Cognitive Flexibility (reaction time)					
Multiple Comparisons					
Time 2	low dose group	Control group	600.73	722.56	0.521
	moderate dose group	Control group	587.12	713.46	
Time 3	low dose group	Control group	608.13	724.59	0.301
	moderate dose group	Control group	605.42	719.90	
Pairwise Comparisons					
Time 2 vs Time 1	low dose group		584.65	711.57	0.215
Time 3 vs Time 1			605.15	723.67	
Time 3 vs Time 2			578.43	710.31	
Time 2 vs Time 1	moderate dose group		574.12	713.98	0.336
Time 3 vs Time 1			595.13	723.52	
Time 3 vs Time 2			578.19	715.59	
Working memory (accuracy)					
Multiple Comparisons					
Time 2	low dose group	Control group	88.05	96.54	0.515
	moderate dose group	Control group	89.46	95.43	
Time 3	low dose group	Control group	88.13	95.30	0.304
	moderate dose group	Control group	88.75	95.15	
Pairwise Comparisons					

Time 2 vs Time 1	low dose group		91.04	96.48	0.314
Time 3 vs Time 1			90.35	94.49	
Time 3 vs Time 2			91.03	95.29	
Time 2 vs Time 1	moderate dose group		91.29	96.23	0.511
Time 3 vs Time 1			90.54	95.23	
Time 3 vs Time 2			91.17	95.89	
Working memory (reaction time)					
Multiple Comparisons					
Time 2	low dose group	Control group	579.59	830.23	0.430
	moderate dose group	Control group	564.12	820.32	
Time 3	low dose group	Control group	578.96	813.57	0.312
	moderate dose group	Control group	575.49	829.32	
Pairwise Comparisons					
Time 2 vs Time 1	low dose group		578.13	834.56	0.31
Time 3 vs Time 1			595.16	821.32	
Time 3 vs Time 2			574.12	823.42	
Time 2 vs Time 1	moderate dose group		567.12	822.15	0.88
Time 3 vs Time 1			578.18	834.67	
Time 3 vs Time 2			568.19	825.19	
